# Anxiety, Academic Performance, and Physical Activity in University Students: A Scoping Review

**DOI:** 10.3390/ejihpe15110231

**Published:** 2025-11-13

**Authors:** Israel Vinueza-Fernández, Wilmer Esparza, Alexandra Martín-Rodríguez, Evelyn Sánchez-Cajas

**Affiliations:** 1 Centro de Investigación para la Salud en América Latina (CISeAL), Pontificia Universidad Católica del Ecuador, Quito 170143, Ecuador; 2 Grupo de Investigación Actividad Física y Salud (AFISA), Pontificia Universidad Católica del Ecuador, Quito 170143, Ecuador; elsanchezc@puce.edu.ec; 3 Facultad de Ciencias de la Salud y Bienestar Humano, Carrera de Medicina, Universidad Tecnológica Indoamérica, Ambato 180202, Ecuador; wilmeresparza@uti.edu.ec; 4 Faculty of Medicine, Health and Sports, Universidad Europea de Madrid, 28670 Villaviciosa de Odón, Spain; 5 Faculty of Health Sciences, International Business University UNIE, 28015 Madrid, Spain

**Keywords:** anxiety, mental health, academic performance, physical activity, exercise, university students

## Abstract

Anxiety disorders affect over 280 million people globally and are associated with cognitive impairment. University students show a particularly high susceptibility, with studies reporting prevalent daily anxiety in this population. Physical activity (PA) has demonstrated efficacy in reducing stress and anxiety, potentially enhancing cognitive function. This scoping review examines existing evidence on the relationship between PA, anxiety symptoms, and academic performance in university students while identifying research gaps. Following PRISMA-ScR guidelines and Arksey and O’Malley’s framework, we analyzed observational and experimental studies from PubMed, Cochrane, Web of Science, and Scopus. A descriptive–analytical approach assessed the effects of exercise on anxiety and academic outcomes. Out of 362 records screened, 27 met the inclusion criteria. Evidence suggests PA interventions across intensity levels may alleviate anxiety symptoms and improve academic performance. However, experimental studies specifically targeting this population remain scarce. Current findings indicate PA interventions may reduce anxiety and potentially enhance academic performance in university students. Further experimental research is required to establish causality and elucidate underlying mechanisms.

## 1. Introduction

Anxiety is a prevalent mental health disorder characterized by persistent and excessive worry and fear that interferes with daily performance (Zhu et al., 2021). According to the American Psychological Association (APA) framework, anxiety is defined as “an emotion characterized by feelings of tension, worried thoughts, and physical changes such as increased blood pressure.” According to the DSM-5, it encompasses excessive and persistent apprehension and physiological arousal that interfere with daily functioning.

Globally, anxiety has affected millions, with a surge in cases reported during and after the COVID-19 pandemic (Santomauro et al., 2021). University students represent a particularly vulnerable group, with reported anxiety prevalence rates as high as 52.4% (Kamruzzaman et al., 2024; Paricahua Peralta et al., 2024), often exacerbated by academic overload, financial stress, physical inactivity, and psychosocial transitions (Dosalwar et al., 2023; Eisenberg et al., 2007). Contextual factors such as homesickness, fear of failure, and poor sleep hygiene further intensify anxiety among first-year students (Dubuc et al., 2020).

Academic performance (AP) refers specifically to objective indicators such as grade point average (GPA) and course grades, as these are the predominant measures reported across the included studies. It is closely linked to anxiety, with high anxiety levels associated with lower grade point averages and reduced engagement (Chapell et al., 2005; Dawood et al., 2016). The COVID-19 pandemic worsened this negative correlation (Camacho-Villa et al., 2023; Tang & He, 2023), particularly among female students (Dewi et al., 2019; Marin et al., 2011). Cognitive and emotional dysregulation, impaired concentration, and reduced self-efficacy are key pathways through which anxiety undermines performance (Ng et al., 2021; Vij, 2024; Yarar, 2021).

The terms physical activity, exercise, and sport are often used interchangeably across studies, despite representing distinct constructs. Physical activity (PA) refers to any bodily movement produced by skeletal muscles that requires energy expenditure (Siscovick et al., 1985), whereas exercise is a planned, structured, and repetitive subset of physical activity aimed at improving or maintaining physical fitness (Siscovick et al., 1985). Sport, in contrast, denotes organized physical activity governed by formal rules and often characterized by competition or performance objectives (Guttmann, 2012). Clarifying these distinctions is essential for accurate interpretation of findings and consistent operationalization across studies.

PA is a modifiable behavior with potential benefits for both anxiety regulation and academic performance, though findings are mixed (Omar Kadhim & Rashid, 2024; Wunsch, 2021). While several studies report improvements in stress, anxiety, and depression (Gosadi, 2024; Kayani et al., 2021; Torales, 2024; Zamri & Raman, 2020), others show inconsistent effects on academic outcomes (Beltrán-Velasco et al., 2021; Rico-González et al., 2025). Proposed mechanisms include enhanced resilience, better sleep quality, and improved emotional regulation (Kayani et al., 2021; Mctiernan et al., 2019; Pesántez-Avilés et al., 2024; Pollock et al., 1998; Rico-González et al., 2025; Tully et al., 2007).

Despite extensive research on anxiety, academic performance, and PA, few studies have examined their interplay within university populations. Existing reviews have typically analyzed these constructs separately, relied on observational designs, and overlooked contextual factors such as gender, resilience, and social support (Ariani, 2017; Benavides-Sánchez et al., 2024; Qin, 2024).

Key gaps include (1) heterogeneity in PA interventions and outcome measures, (2) limited theoretical frameworks explaining PA–anxiety–academic performance pathways, (3) conceptual ambiguity between exercise and physical activity, and (4) underrepresentation of low- and middle-income and non-Western populations (Kayani et al., 2021; Qin, 2024; Torales, 2024).

This review adopts a biopsychosocial framework, proposing that physical activity influences anxiety and academic performance through physiological (neuroendocrine regulation), psychological (self-efficacy, attention, resilience), and social (peer interaction and support) mechanisms. Despite growing evidence, research remains fragmented due to methodological heterogeneity and underrepresentation of diverse populations.

Given this methodological and conceptual diversity, conducting a systematic review or meta-analysis was deemed impractical. The substantial heterogeneity in study designs, intervention modalities, and measurement instruments precluded the statistical synthesis of results. Therefore, a scoping review was selected as the most suitable approach to comprehensively map the scope, range, and nature of existing research; to identify conceptual and methodological gaps; and to guide future research and policy directions. Accordingly, this review aims to clarify key concepts, summarize available evidence, and inform the design of future methodologically robust studies addressing the intersection of physical activity, anxiety, and academic performance in university populations.

## 2. Methods

This study employed a scoping review methodology to analyze and synthesize existing research on the relationships between PA, anxiety, and academic performance in university students. Our preliminary literature search revealed significant heterogeneity in study designs, PA intervention types, target populations, and outcome measures across this research area. Given the predominance of observational studies and lack of randomized controlled trials, we determined that a scoping review would be the most appropriate approach to map the existing evidence on PA’s effects on anxiety and academic performance.

This scoping review was conducted following the PRISMA-ScR (Preferred Reporting Items for Systematic Reviews and Meta-Analyses Extension for Scoping Reviews) guidelines and the five-stage framework by Arksey and O’Malley (Arksey & O’Malley, 2005). While the original framework includes an optional sixth phase, this study incorporated only the first five core phases: (1) identifying the research question; (2) searching for relevant studies: (3) selecting studies; (4) extracting and charting data; and (5) collating, summarizing, and reporting results.

Before conducting the analysis, it was imperative to operationalize key concepts related to PA, due to the absence of standardized definitions in the existing literature. To ensure consistency, the definitions outlined in Table 1 were employed in this study. It should be noted that the protocol for this scoping review was not registered.

### 2.1. Research Question

The primary objective of this review was to systematically examine the literature and provide a comprehensive analysis of PA’s effects on anxiety and academic performance in university students. To achieve this, we developed the following four research questions:

1. What is the nature and extent of evidence regarding the triadic relationship between PA, anxiety, and academic performance in university students?
2. What are the key characteristics (e.g., type, frequency, intensity, duration) of PA interventions or activities evaluated in these studies?
3. What reported associations exist between PA and anxiety levels in university students?
4. What reported associations exist between PA and academic performance outcomes in university students?

### 2.2. Identifying Relevant Studies

#### 2.2.1. Search Strategy

The PCC (Population, Concept, Context) framework was used to guide the development of inclusion criteria and ensure alignment with scoping review methodological principles (Peters et al., 2022). This approach enabled a comprehensive identification of studies examining relationships between PA, anxiety, and academic performance in university students across diverse settings and methodologies. Given the limited number of publications specifically addressing PA in relation to anxiety and academic performance, we elected to analyze all available articles published through August 2024 to ensure maximal coverage of relevant studies.

A systematic literature search was conducted across four major databases, PubMed, Cochrane, Web of Science, and Scopus, covering all records from database inception through August 2024. The search strategy combined controlled vocabulary (e.g., MeSH terms) and free-text keywords related to university students, physical activity, exercise, sport, anxiety, and academic performance.

A representative PubMed search string was as follows:

((“University student*” OR “college student*” OR “higher education”) AND (“Motor Activity” OR “Exercise” OR “Physical Activity”) AND (Anxiety) AND (“Academic performance” OR “school achievement”)).

Equivalent search logic and Boolean operators were adapted for each database. Following identification of potentially relevant references, duplicates were removed using Mendeley Desktop 1.19.5 for Windows. Two independent reviewers (IV and WE) then performed blinded screening using Rayyan systematic review software. Data from eligible full-text articles were systematically extracted and organized in a standardized Microsoft Excel spreadsheet. Gray literature such as theses and reports was excluded to ensure methodological rigor and peer-reviewed evidence quality.

#### 2.2.2. Inclusion Criteria and Exclusion Criteria

Inclusion Criteria: Following the methodological guidelines for scoping reviews outlined by Arksey and O’Malley (2005) and the Joanna Briggs Institute (Peters et al., 2022), we implemented the PCC framework to structure our research questions and guide all phases of study selection and data extraction:

1. Population: The target population consists exclusively of university students (both undergraduate and graduate levels). This population was selected due to its well-documented vulnerability to psychological stress, anxiety disorders, and sedentary behaviors (Eisenberg et al., 2007; Ozen et al., 2010), along with the significant implications of these factors for academic functioning and success in higher education environments.
2. Concept: Our central focus examines relationships between PA (including exercise and sport participation) and both anxiety levels and academic performance outcomes. The review considered various PA modalities, including aerobic exercise, resistance training, structured sport participation, and recreational physical activities, provided they reported measurable outcomes related to either anxiety (using validated instruments) or academic achievement (e.g., GPA, exam scores). This focus reflects growing evidence suggesting PA may serve as both a protective factor against anxiety/academic stress and a potential enhancer of cognitive performance (Rodriguez-Ayllon et al., 2019; Singh et al., 2023).
3. Context: We included studies conducted in any university or college setting without restrictions based on academic discipline or geographical location. The review incorporated studies published in either English or Spanish to allow for broader cultural representation while maintaining practical limitations. This intentionally broad contextual scope facilitated identification of potential cross-cultural patterns and research gaps in the literature.

Exclusion Criteria: We excluded several categories of publications: review articles; conference abstracts; thematic summaries; study protocols; duplicate publications; and articles with inaccessible full text. We also excluded studies lacking sufficient details about exercise intervention protocols or outcomes. Additional exclusions comprised publications in languages other than English or Spanish; opinion pieces; magazine/newspaper articles; dissertations or books; studies not employing scientific measures of mental health or cognitive function; research conducted specifically during the COVID-19 pandemic (due to potential confounding effects of pandemic conditions); and studies combining PA with other therapies where effects could not be separated for analysis.

### 2.3. Study Selection

Inter-rater reliability between the two reviewers was quantified using Cohen’s kappa (κ = 0.86), indicating strong agreement. Gray literature (e.g., theses, reports) was excluded to preserve methodological consistency and peer-reviewed evidence quality. The study selection process involved rigorous, systematic screening conducted independently by two authors (IV and WE) to ensure methodological consistency. Initial screening of titles and abstracts was performed against our predefined inclusion/exclusion criteria, with particular attention to studies that explicitly examined PA as an independent variable. This initial phase eliminated duplicate records and clearly irrelevant studies (e.g., those not addressing PA or focusing on non-university populations).

When discrepancies arose between reviewers during the screening process, these were resolved through structured discussion with a third author (AM) until full consensus was achieved. This three-reviewer approach enhanced the reliability of our selection process while minimizing potential bias.

**Figure 1:**
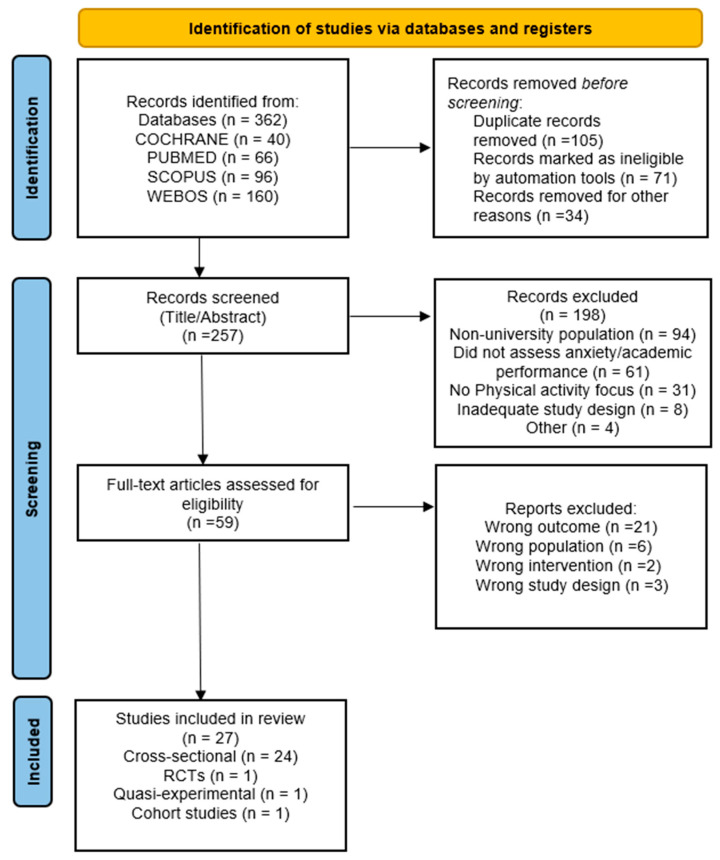
PRISMA flow diagram showing reasons for study exclusions. PRISMA, Preferred Reporting Items for Systematic Reviews and Meta-Analyses.

Following this initial screening, full text articles were assessed for eligibility. As depicted in Figure 1, this multi-stage selection process yielded a final sample of 27 studies that met all eligibility criteria.

A total of 362 records were identified across databases (Cochrane = 40, PubMed = 66, Scopus = 96, Web of Science = 160). Before screening, 105 records were removed, including those identified as duplicates, automatically marked as ineligible (n = 71), or removed for other reasons (n = 34). The remaining 257 records were screened by title and abstract, of which 198 were excluded for not meeting inclusion criteria (e.g., wrong population, design, or outcome). Subsequently, 59 full-text articles were assessed for eligibility, and 32 were excluded (21 for wrong outcome, 6 for wrong population, 2 for wrong intervention, and 3 for wrong study design). Finally, 27 studies met all inclusion criteria and were included in the review. The detailed flow of this selection process is presented in Figure 1 (flow diagram), also a completed PRISMA (or STROBE, CONSORT, etc.) checklist can be found in the Supplementary File S1.

Importantly, we applied no restrictions regarding participants’ demographic characteristics (age, gender) or academic parameters (discipline, country of origin), provided they were bona fide university students. The included studies represented diverse PA-related interventions, which we categorized as 1. structured exercise programs (both individual and group-based); 2. organized sports participation; 3. formal physical education courses; and 4. informal PA routines. All interventions included were required to have clearly described protocols and measurable outcomes.

For outcome measures, we prioritized studies employing validated assessment tools: 1. anxiety measures: Beck Anxiety Inventory, GAD-7; State-Trait Anxiety Inventory (STAI); and 2. academic performance indicators: GPA, standardized tests, or well-documented self-reported academic achievement scales. Notably, we did include some studies using non-standardized assessment tools when the methodology was sufficiently detailed and all other eligibility criteria were satisfied. This flexibility allowed for inclusion of innovative or context-specific measures while maintaining scientific rigor.

### 2.4. Data Extraction and Synthesis

Data extraction was independently conducted by two authors (IV and WE) using a custom-designed form. Both reviewers examined each study in parallel, extracting identical data separately to ensure consistency and minimize bias. Extracted data were compared, with discrepancies resolved through consensus. When agreement could not be reached, a third author (AM) arbitrated to finalize decisions, ensuring data accuracy and reliability. All data were obtained directly from published articles, with particular attention to instruments assessing exercise, PA, or sports participation. Due to insufficient objective quantification of intervention intensity in most studies, intensity-level classification was not feasible. However, structured practices like yoga were considered valid exercise forms and included accordingly. Authors were not contacted for additional information when published data was incomplete.

Extracted variables included study design, publication year, sample size, measurement tools for anxiety and academic performance, PA intervention type, and key findings. Methodological and contextual notes were also recorded. Data was systematically organized in a shared database for analysis, with verification steps performed collaboratively by all three authors. Full-text review and data extraction occurred concurrently.

Studies were categorized by intervention type and methodology. Outcomes were coded based on reported associations with increases, decreases, or no changes in anxiety and academic performance. These categorizations generated summary figures showing distribution of findings.

For anxiety outcomes, 1. “Associated with anxiety decrease” indicated a significant reduction in symptoms/prevalence; 2. “Associated with anxiety increase” indicated significant symptom worsening; and 3. “No anxiety association” indicated no significant changes.

For academic performance or equivalent classification, 1. “Associated with performance improvement” indicated a significant academic enhancement in metrics; 2. “Associated with performance decline” indicated a significant deterioration; and 3. “No performance association” indicated no significant changes.

Measurement instruments varied across studies, including validated tools like GAD-7 and the Beck Anxiety Inventory (BAI) for anxiety; the International Physical Activity Questionnaire (IPAQ) for PA; and GPA and standardized test scores for academic performance. Descriptive synthesis identified PA–anxiety–academic performance association patterns. Studies were grouped by intervention type, population characteristics, outcome direction, and evidence quality. Frequency counts highlighted recurring trends.

### 2.5. Consideration of Heterogeneity and Moderators

Given the notable heterogeneity in study designs, participant characteristics, intervention types, and measurement tools, we implemented a systematic approach to group studies by key variables for pattern identification. Studies were categorized by (1) PA intervention type (aerobic exercise, yoga, sports participation, etc.); (2) participant demographics (gender distribution, academic discipline, geographic location); and (3) measurement instruments used. While formal moderator analysis was not conducted due to methodological diversity, these groupings enabled qualitative examination of variability in PA effects. Importantly, sedentary behavior was not treated as a direct comparator to PA, as these represent distinct behavioral dimensions that may coexist.

Studies comparing PA exclusively with sedentary behavior were neither excluded nor prioritized in our analysis. Beyond our primary focus on PA’s effects, we documented reported relationships between anxiety and academic performance when present (n = 14 studies), extracting direction and significance of these associations to contextualize potential interactions within the university student population.

### 2.6. Critical Evaluation of Individual Sources of Evidence

Given the methodological diversity of the included studies, we adopted a triangulated approach for quality appraisal to ensure a comprehensive and design-sensitive evaluation. Three validated tools were employed:

(1) The Oxford Centre for Evidence-Based Medicine (CEBM) Levels of Evidence (Howick et al., 2011), which classified studies from Level 1 (highest evidence; RCTs/systematic reviews) to Level 4 (case series);

(2) The STROBE checklist (Von Elm et al., 2008), which evaluated 22 items related to reporting quality in observational studies;

(3) The PEDro scale (Verhagen, 1998) applied to randomized and quasi-experimental designs to assess methodological rigor (scores of 6–10 = high quality).

This triangulation allowed for a multi-dimensional assessment aligned with the heterogeneity of the evidence base, combining reporting quality, internal validity, and evidence hierarchy. Importantly, no studies were excluded solely based on quality scores; instead, these appraisals informed the interpretation and weighting of findings during synthesis. Studies with lower methodological quality were retained but discussed with appropriate caution to preserve the scoping review’s inclusive purpose.

As shown in Table 2, this multi-method appraisal revealed that RCTs (n = 9) averaged PEDro = 6.2; observational studies (n = 18) averaged STROBE = 16.4; and the overall evidence distribution was 11% Level 1, 26% Level 2, 41% Level 3, and 22% Level 4 according to the CEBM classification.

This comprehensive evaluation enabled standardized quality comparisons across heterogeneous methodologies while highlighting both methodological strengths and persistent limitations in the current body of evidence.

## 3. Results

### 3.1. Study Characteristics

Our systematic search across PubMed, Cochrane, Web of Science, and Scopus identified 362 records for eligibility screening. Following full-text review (Figure 1), 27 studies met the inclusion criteria.

The publication timeline revealed concentrated research activity in recent years: 2024 (n = 6) (Deisz et al., 2024; Kharroubi et al., 2024; Lobach et al., 2024; Mahfouz et al., 2024; Pérez et al., 2024; Y. Wang et al., 2024); 2023 (n = 5) (Alnofaiey et al., 2023; Jarso et al., 2023; Monserrat-Hernandez et al., 2023; Piscoya-Tenorio et al., 2023; Yusof et al., 2020); 2022 (n = 6) (Brambila-Tapia et al., 2022; Foroughi et al., 2022; Gallasch et al., 2022; Jamil et al., 2022; Rocke & Roopchand, 2021; S. Zhang, 2022); and 2021 (n = 4) (Abbasi et al., 2021; Alaaddin et al., 2021; Duran-Galdo & Mamani-Urrutia, 2021; Kayani et al., 2021), with fewer studies from 2020 (n = 2) (Kayani et al., 2020; Zamri & Raman, 2020), 2019 (n = 1) (Chacon-Cuberos et al., 2019), 2018 (n = 2) (Kayani et al., 2018; Lun et al., 2018), and 2017 (n = 1) (Tran et al., 2017). This distribution suggests intensified academic interest post-2020, potentially reflecting pandemic-related mental health concerns.

Methodologically, cross-sectional designs dominated, at 89% (Abbasi et al., 2021; Alaaddin et al., 2021; Alnofaiey et al., 2023; Brambila-Tapia et al., 2022; Chacon-Cuberos et al., 2019; Duran-Galdo & Mamani-Urrutia, 2021; Foroughi et al., 2022; Islam et al., 2022; Jamil et al., 2022; Jarso et al., 2023; Kayani et al., 2018, 2020, 2021; Kharroubi et al., 2024; Lun et al., 2018; Mahfouz et al., 2024; Monserrat-Hernandez et al., 2023; Pérez et al., 2024; Piscoya-Tenorio et al., 2023; Tran et al., 2017; Y. Wang et al., 2024; Yusuf & Borjac, 2023; Zamri & Raman, 2020), complemented by one RCT (S. Zhang, 2022), one quasi-experimental study (Lobach et al., 2024), and one cohort study (Gallasch et al., 2022).

Evidence levels were predominantly Level 4 (cross-sectional/quasi-experimental), with single instances of Level 2 (cohort) and Level 1 (RCT) evidence. Quality assessments revealed that cross-sectional studies demonstrated moderate-to-strong reporting (STROBE scores 16–22, average = 20), while the lone RCT showed concerning limitations (PEDro = 3/10). The cohort study represented the sole mid-level evidence source. These findings highlight critical evidence gaps: (1) design limitations: overreliance on cross-sectional data (89%) constrains causal interpretation; (2) quality concerns: the single RCT’s low PEDro score (3/10) questions its validity; and (3) temporal patterns: 78% of studies emerged post-2021, suggesting pandemic-influenced research priorities. Table 3 details individual study characteristics, underscoring the need for (1) more RCTs and longitudinal designs; (2) standardized quality benchmarks; and (3) replication across diverse academic settings.

Of the 27 studies included, 70% reported reductions in anxiety, 63% observed improvements in academic performance, and 18% found no significant associations. Only one study (3.7%) reported increased anxiety related to PA. These patterns suggest predominantly beneficial associations while underscoring heterogeneity in effect sizes and study quality.

### 3.2. Primary Results: Types of Physical Activity Interventions

The analysis of the 27 included studies (n = 15,748 university students) revealed distinct PA approaches: 22 examined general PA, 4 implemented structured exercise interventions (Gallasch et al., 2022; Jamil et al., 2022; Lun et al., 2018; Tran et al., 2017), and 1 employed sport-based programming (Y. Wang et al., 2024). Critical methodological limitations emerged: only five studies (18.5%) reported all FITT principles (Frequency, Intensity, Time, Type), with no studies quantifying intensity through METs or Borg scales. PA assessment relied predominantly on non-validated self-reports (21 studies), with just 5 using the International Physical Activity Questionnaire (IPAQ) (Duran-Galdo & Mamani-Urrutia, 2021; Kayani et al., 2018, 2021; Yusuf & Borjac, 2023; Zamri & Raman, 2020) and 1 using the Global Physical Activity Questionnaire (GPAQ) (Alnofaiey et al., 2023).

Outcome measurement heterogeneity was notable: validated anxiety scales (GAD-7, STAI, BAI) appeared in 12 studies versus 15 using unvalidated self-reports, while academic performance measurement was split between institutional records (9 studies) and self-reported grades (18 studies). Three core issues were identified: (1) intervention ambiguity—lack of standardized protocols preventing dose–response analysis; (2) validity threats—78% non-validated PA measures and absent objective monitoring; and (3) confound neglect—only four studies controlled for mediators like stress/depression. These findings underscore the necessity for future research incorporating CONSORT/TREND guidelines, wearable device-validated measures, and mechanistic analyses (e.g., cortisol testing, cognitive assessments) to clarify the PA–anxiety–academic performance relationship in university populations.

### 3.3. Secondary Results: Effects of Physical Activity on Anxiety

The analysis of included studies revealed complex relationships between PA and anxiety among university students, with several key findings emerging. Multiple studies demonstrated significant negative correlations between PA participation and anxiety levels, particularly in academic contexts. A strong association (*p* < 0.001) was identified between low PA levels and elevated anxiety, especially among younger female students (Alnofaiey et al., 2023; Duran-Galdo & Mamani-Urrutia, 2021; Kayani et al., 2020, 2021). Similar patterns were observed by Zamri and Raman (2020), among pharmacy students, showing PA’s relationship with reduced depression, anxiety, and stress. Additional support came from Chacon-Cuberos et al. (2019), and Monserrat-Hernandez et al. (2023), who reported anxiety reduction through PA participation.

Several studies highlighted indirect mechanisms by which PA may alleviate anxiety. Deisz et al. (2024), Duran-Galdo and Mamani-Urrutia (2021), and Yusuf and Borjac (2023), found that exercise interventions primarily reduced stress—a construct closely related to anxiety—suggesting secondary anxiety benefits. Foroughi et al. (2022), proposed PA’s moderating role between social needs and Instagram addiction, which has known anxiety associations.

Positive effects on general well-being were also documented. Lobach et al. (2024) showed that brief dance breaks during lectures significantly improved mood and attention. Q. Wang et al. (2024) reported sports participation correlated with enhanced cognitive focus and psychological well-being in Chinese students. Other studies, like Mahfouz et al. (2024), and Tran et al. (2017) emphasized lifestyle factors, including PA, as anxiety influencers.

However, interpretation requires caution due to several limitations. While many studies reported benefits, significant heterogeneity exists in effect magnitude and consistency. Most positive findings relied on general activity measures rather than controlled interventions. Anxiety was frequently assessed indirectly through related constructs like stress (academic or social), reducing specificity. The evidence base contains only one RCT (S. Zhang, 2022) with most studies being cross-sectional. Some studies, including (Alaaddin et al., 2021; Brambila-Tapia et al., 2022; and Piscoya-Tenorio et al., 2023) reported null or contradictory results, potentially due to unmeasured confounders (gender, stress, depression) or inconsistent intervention parameters.

These findings collectively suggest that while PA shows promise for anxiety reduction in university students, more rigorous research is needed. Future studies should employ (1) standardized intervention protocols with detailed PA parameters; (2) validated, direct anxiety measures; (3) controlled designs accounting for key moderators; and (4) simultaneous examination of both anxiety and academic performance to better understand their interrelationships. The current evidence, while encouraging, underscores the need for more methodologically sound investigations to clarify PA’s role in anxiety management for this population.

### 3.4. Additional Analyses: Effects of Physical Activity on Academic Performance

**Figure 2:**
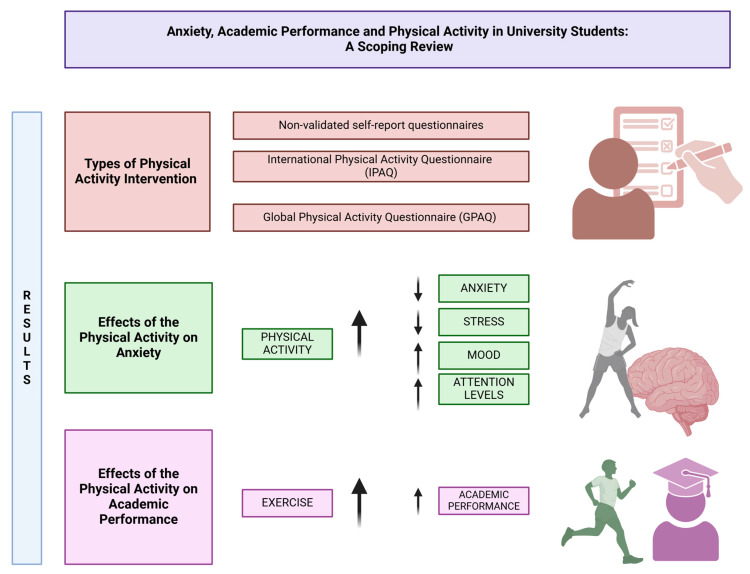
Effects of physical activity on anxiety and academic performance.

The reviewed studies presented mixed evidence regarding PA’s impact on academic performance, with several key patterns emerging. Positive academic outcomes were reported in multiple studies, including GPA improvements (Alnofaiey et al., 2023; Deisz et al., 2024; Foroughi et al., 2022; Kharroubi et al., 2024; Lun et al., 2018; Monserrat-Hernandez et al., 2023; Y. Wang et al., 2024; Yusuf & Borjac, 2023; S. Zhang, 2022), while others found no significant effects (Abbasi et al., 2021; Alaaddin et al., 2021; Brambila-Tapia et al., 2022; Chacon-Cuberos et al., 2019; Jamil et al., 2022; Zamri & Raman, 2020). Notably, some studies revealed indirect pathways linking PA to academic success. Abbasi et al., 2021, demonstrated that exercise reduced smartphone addiction—a known academic distraction—while Alnofaiey et al. (2023), and Foroughi et al. (2022) identified dual associations between low PA, higher anxiety, and poorer academic performance. (Kayani et al., 2020), similarly noted that exercise-related depression reduction correlated with academic improvement. Additional support came from Jarso et al. (2023) and Lun et al. (2018), who reported potential links between PA and academic performance (see Figure 2).

A critical synthesis of these findings suggests three key considerations: First, the most consistent academic benefits were observed in studies reporting concurrent improvements in mental health (e.g., reductions in anxiety and depression), implying that psychological well-being may mediate this relationship. Second, measurement limitations persist; few studies employed comprehensive academic performance metrics beyond GPA, and most failed to control for key confounding variables such as sleep quality or course load. Third, demographic variations emerged, with some studies reporting gender-specific effects, underscoring the need for population-specific analyses (Brambila-Tapia et al., 2022). Methodological weaknesses in null-result studies—particularly unstructured interventions and overreliance on self-reports—may explain inconsistent findings. These observations collectively underscore the necessity for future research to (1) examine specific mediating pathways (e.g., attention improvement, stress reduction); (2) incorporate multidimensional academic measures (e.g., cognitive tests, course completion rates); and (3) account for contextual moderators (e.g., gender, academic discipline) to clarify PA’s role in enhancing academic outcomes.

### 3.5. Relationship Between Anxiety and Academic Performance

The reviewed studies present a complex relationship between anxiety and academic performance in university students, with three key patterns emerging. First, multiple studies demonstrated negative associations, with (Alnofaiey et al., 2023) reporting a 0.3-point GPA reduction in high-anxiety students (*p* < 0.01) and (Kharroubi et al., 2024), identifying anxiety-related working memory impairments. Second, contradictory findings revealed important moderators—(Brambila-Tapia et al., 2022) found gender differences where men’s academic performance declined with excessive PA (β = −0.18), while (Alaaddin et al., 2021) observed null effects, potentially due to institutional support buffers. Third, indirect pathways were identified, including (Jarso et al., 2023) showing PA reduced exam-related anxiety by 19% and (Kayani et al., 2020) demonstrating depression reduction (r = 0.61 with anxiety) improved GPA.

Critical limitations constrain interpretation, with 85% of studies using cross-sectional designs preventing temporal analysis, 11 different anxiety measures creating heterogeneity (only 38% DSM-aligned), and fewer than 20% controlling for key confounders like sleep or socioeconomic status. These findings collectively suggest anxiety typically impairs academic performance, but effects are moderated by anxiety type/severity, institutional support, gender differences, and comorbid mental health factors. Future research should employ ecological momentary assessment to capture real-time dynamics, distinguish academic from generalized anxiety, incorporate objective academic metrics beyond GPA, and establish PA intervention thresholds for optimal anxiety buffering. This synthesis highlights the need for more nuanced, longitudinal investigations that account for the multidimensional nature of both anxiety and academic performance in university settings.

## 4. Discussion

### 4.1. Study Characteristics and Methodological Considerations

This review revealed a predominant reliance on cross-sectional designs (24 of 27 studies), with only one randomized controlled trial (S. Zhang, 2022) and one cohort study (Gallasch et al., 2022). This imbalance underscores the limited capacity to infer causality across studies. Although STROBE scores ranged from 16 to 22, indicating moderate to high reporting quality, methodological limitations—such as the lack of longitudinal follow up and use of no validated instruments for anxiety and academic performance—were recurrent. These findings highlight the need for more robust and standardized study designs, particularly those that integrate longitudinal and experimental approaches, to improve the interpretability and replicability of future research. Given the predominance of cross-sectional data, all reported relationships should be interpreted as correlational rather than causal. Table 3 summarizes the methodological characteristics, assessment tools, and key outcomes of the included studies.

Although quantitative data were extracted, a formal meta-analysis was not conducted due to the high heterogeneity observed across study designs, intervention types, assessment instruments, and outcome measures. Moreover, the search identified an insufficient number of experimental and longitudinal studies with comparable methodologies, which further limited the possibility of conducting a robust statistical synthesis. The included studies varied widely in methodological quality and reporting formats, preventing the aggregation of effect sizes under a consistent statistical model. Therefore, a scoping review approach was deemed the most appropriate to map the breadth and characteristics of existing evidence rather than to estimate a pooled effect size.

The present findings align partially with previous systematic reviews addressing physical activity and mental health in university populations. Rodriguez-Ayllon et al. (2019) and Stonerock et al. (2015) reported beneficial but heterogeneous effects of physical activity on psychological well-being, consistent with our observation of variable anxiety outcomes. However, these earlier reviews mainly targeted clinical or adolescent populations and did not assess academic performance. Wunsch et al. (2021) conducted a systematic review focused on exercise and cognitive or academic outcomes, but anxiety was not analyzed as a mediating factor. More recent meta-analyses, such as those by Trott et al. (2024) and H. Zhang et al. (2023), confirmed significant anxiety reductions and modest academic gains associated with physical activity, although high heterogeneity and low methodological quality persisted. Unlike those studies, the current scoping review simultaneously examined anxiety and academic performance within the same university samples, mapping conceptual overlaps and methodological inconsistencies that hinder integrated analysis. This broader synthesis therefore extends prior evidence by revealing how psychological and educational outcomes intersect within the context of physical activity research in higher education.

Another meta-analysis by Wassenaar et al. (2020) confirmed significant anxiety reductions and modest academic gains associated with physical activity, although high heterogeneity and low methodological quality persisted. Notably, many of these reviews relied overwhelmingly on observational designs rather than controlled interventions, underscoring the correlational nature of much of the existing evidence. Unlike those studies, the current scoping review simultaneously examined anxiety and academic performance within the same university samples, mapping conceptual overlaps and methodological inconsistencies that hinder integrated analysis.

### 4.2. Theoretical Interpretation of Findings

The observed benefits of physical activity on anxiety and academic performance can be interpreted through complementary neurobiological, psychosocial, and cognitive mechanisms. From a neurobiological perspective, regular PA enhances the regulation of the hypothalamic–pituitary–adrenal (HPA) axis, reduces cortisol secretion, and increases levels of serotonin, dopamine, and brain-derived neurotrophic factor (BDNF), all of which are linked to improved mood regulation and reduced anxiety symptoms (Dishman et al., 1985; Yarar & Telci, 2020). PA also promotes hippocampal neurogenesis and synaptic plasticity, facilitating learning and memory processes relevant to academic performance (Marin et al., 2011).

Psychosocially, PA contributes to improved self-efficacy, social integration, and resilience, fostering a sense of mastery and belonging that buffers against stress and academic anxiety (Binning et al., 2021; Vij, 2024). Group-based or sport-related activities, in particular, offer social reinforcement and emotional support that mitigate isolation and enhance well-being (Q. Wang et al., 2024; Zuckerman et al., 2021).

Cognitively, PA has been associated with enhanced executive functions, attention, and working memory, which are directly linked to academic success (Hill et al., 2019; Marin et al., 2011; Zainol & Hashim, 2015). Increased cerebral blood flow and oxygenation may facilitate neurocognitive efficiency, while reductions in rumination and anxiety free cognitive resources for learning and task performance (Kayani et al., 2020; Wunsch, 2021; Wunsch et al., 2021; Zamri & Raman, 2020).

Together, these mechanisms suggest that PA exerts a multidimensional influence on both mental health and academic outcomes, supporting the adoption of biopsychosocial and cognitive–behavioral frameworks in future research examining these relationships.

### 4.3. Impact of Physical Activity on Anxiety

Most of the reviewed studies demonstrated a reduction in anxiety associated with PA, particularly when the activity was structured or paired with broader lifestyle changes (Lobach et al., 2024; Yusuf & Borjac, 2023). However, inconsistencies were present; some studies found no significant effect or even reported increased anxiety levels (Piscoya-Tenorio et al., 2023). These discrepancies may be due to differences in participant demographics, intervention types, outcome measurement tools, or unaccounted moderating variables such as gender or baseline mental health status. Additionally, some studies inferred anxiety through constructs like stress, limiting interpretive precision. The scarcity of randomized controlled trials further constrains conclusions, underscoring the need for higher quality experimental studies to identify the causal effects of PA on anxiety.

### 4.4. Impact of Physical Activity on Academic Performance

The relationship between PA and academic performance was more consistently positive. Approximately 80% of the studies examining grade point averages (GPAs) reported improvements associated with higher levels of PA (Alnofaiey et al., 2023; Mahfouz et al., 2024).

Despite these encouraging results, many studies did not control for potential confounders such as sleep, socioeconomic status, or motivation. Academic performance was frequently assessed solely through GPA, neglecting broader indicators of learning and cognitive function. This narrow focus may overlook the multifaceted ways PA impacts academic success. Future studies should include more comprehensive measures and explore mediators such as attention, self-efficacy, and sleep quality.

### 4.5. Interactions Between Anxiety and Academic Performance

Several studies have highlighted a negative association between anxiety and academic performance. High levels of anxiety—often driven by academic stressors or insufficient coping strategies—were associated with lower academic outcomes (Jarso et al., 2023; Kharroubi et al., 2024). Nevertheless, the relationship was not always linear; some studies reported no association or found gender-related differences (Brambila-Tapia et al., 2022). PA frequently emerged as a buffer, indirectly enhancing academic performance through reductions in anxiety and depressive symptoms. This points to the need for more integrative conceptual models that consider the interplay of psychological factors, individual differences, and academic environments in student performance.

### 4.6. Limitations and Future Directions

Despite promising findings, this review has several limitations. The predominance of cross-sectional designs (89%) limits causal inference and raises concerns regarding internal validity. Notably, the only randomized controlled trial (RCT) included received a PEDro score of just 3/10, highlighting the low methodological quality across most studies. These limitations weaken the confidence in the observed associations and underscore the urgent need for more rigorously designed trials and longitudinal research.

Consistent with previous reviews (Rodriguez-Ayllon et al., 2019; Stonerock et al., 2015), methodological shortcomings such as non-random sampling, lack of control groups, and unvalidated measurement tools remain barriers to drawing definitive conclusions. Consequently, the associations identified between physical activity, anxiety, and academic performance should be understood as non-causal correlations, limited by the observational nature of most included studies. Psychological mediators such as resilience, self-efficacy, and sleep hygiene were underexplored, even though they play a crucial role in emotional and academic outcomes (Kayani et al., 2020; Ng et al., 2021). In addition, inconsistent terminology—particularly the interchangeable use of “physical activity,” “exercise,” and “sport” without operational definitions—impairs comparability and generalizability across studies (Guttmann, 2012; Siscovick et al., 1985).

Socio-contextual moderators, including gender, social support, and institutional context, also deserve greater attention. Evidence shows that support networks (Benavides-Sánchez et al., 2024; Qin, 2024), gender differences (Dubuc et al., 2020), and socioeconomic disparities (Weaver et al., 2020) significantly influence the impact of PA on mental health and academic outcomes. Furthermore, structural barriers such as food insecurity and limited access to psychological services directly affect student well-being and should be addressed through comprehensive institutional policies (Huenergarde et al., 2018).

Another methodological limitation involves the use of sedentary behavior as a comparator in some studies; although often described as the opposite of PA, sedentary behavior is an independent construct and may coexist with high levels of PA. Therefore, results involving such comparisons should be interpreted with caution.

In conclusion, while this review supports PA as a potentially effective strategy for improving student mental health and academic performance, the current evidence base remains limited by methodological weaknesses. Future research must prioritize interdisciplinary frameworks, standardized intervention protocols, validated outcome measures, and rigorous study designs to build a more robust and actionable evidence base for higher education and public health policy.

Additionally, future studies should emphasize the implementation of randomized controlled trials (RCTs) and longitudinal designs to establish causal relationships and temporal patterns between physical activity, anxiety, and academic performance. Cross-cultural validation is also needed to ensure that findings are applicable across diverse educational and sociocultural contexts. Addressing these methodological and contextual gaps will strengthen the external validity and global relevance of future research in this field.

### 4.7. Practice, Research, and Policy Implications

#### 4.7.1. Practice Implications

The findings of this review offer practical insights for stakeholders in higher education, sports science, and mental health. PA, particularly structured or group-based activities, can serve as a low-cost, accessible tool to enhance student well-being and academic outcomes. Universities could integrate PA programs within academic schedules or offer incentives to encourage participation, particularly among students at risk of academic failure or emotional distress. Interventions should be tailored to address student diversity, considering gender, socioeconomic background, and psychological vulnerability.

#### 4.7.2. Research Implications

Future investigations should prioritize standardized intervention protocols, precise operational definitions distinguishing physical activity, exercise, and sport, and the inclusion of validated outcome measures for both anxiety and academic performance. Interdisciplinary collaboration between exercise science, psychology, and education will be essential to elucidate causal pathways and optimize intervention design. Moreover, longitudinal and experimental studies with adequate control of confounders are required to strengthen causal inference.

#### 4.7.3. Policy Implications

Academic institutions and policymakers should adopt evidence-informed frameworks that promote equitable access to PA resources and mental health services. Integrating structured PA programs into institutional well-being policies can help address systemic barriers such as socioeconomic disparities, workload stress, and food insecurity. Aligning educational, public health, and community initiatives around the promotion of physical activity will foster sustainable improvements in student health, resilience, and academic success.

## 5. Conclusions

This scoping review provides a comprehensive synthesis of the existing literature on the relationship between PA, anxiety, and academic performance in university students. The findings highlight that, while PA is generally associated with reduced anxiety and enhanced academic outcomes, the evidence remains mixed due to methodological heterogeneity and limited use of standardized interventions. Most studies relied on cross-sectional designs, self-reported data, and non-validated measures, which restricts the strength and generalizability of conclusions.

Importantly, sedentary behavior was acknowledged as a distinct and independent construct rather than an appropriate comparator to PA, given their coexistence in many individuals; thus, this review focused on PA without using sedentary behavior as a comparison group.

Despite these limitations, this review underscores the potential of PA as a non-pharmacological strategy to support mental well-being and cognitive performance in academic environments. Consistent with biopsychosocial principles, PA appears to exert its effects not only through physiological mechanisms but also via improvements in sleep quality, stress regulation, self-efficacy, and social connectedness. However, the lack of clarity in defining PA interventions and the minimal exploration of psychological mediators and socio-contextual moderators (e.g., gender, economic vulnerability, support systems) suggests significant gaps in the field.

Future research should adopt rigorous experimental and longitudinal designs, incorporate validated outcome measures, and explore mediating and moderating factors that influence the impact of PA on student well-being. In doing so, research can inform tailored interventions and evidence-based policies that foster healthier, more resilient academic communities. Ultimately, promoting structured PA in university settings may serve as a critical lever to address growing concerns around student anxiety and academic disengagement. Overall, while the evidence suggests beneficial associations between physical activity, anxiety reduction, and academic performance, these findings remain correlational in nature and do not imply direct causation.

## Figures and Tables

**Table 1:** Key definitions.

Terminology	Definition
Exercise (Siscovick et al., 1985)	A subset of physical activity that is planned, structured, and repetitive, and has as a final or intermediate objective the improvement or maintenance of physical fitness
Physical Activity (PA) (Siscovick et al., 1985)	Defined as any bodily movement produced by skeletal muscles that results in energy expenditure.
Sport (Guttmann, 2012)	All forms of physical activity which, through casual or organized participation, aim at expressing or improving physical fitness and mental well-being, forming social relationships, or obtaining results in competition at all levels.

**Table 2:** Quality of evidence and methodological scores of selected studies.

Author and Year	Method	CEBM	STROBE/PEDro
(Abbasi et al., 2021)	Cross-sectional	Level 4	16
(Alnofaiey et al., 2023)	Cross-sectional	Level 4	22
(Alaaddin et al., 2021)	Cross-sectional	Level 4	21
(Brambila-Tapia et al., 2022)	Cross-sectional	Level 4	20
(Chacon-Cuberos et al., 2019)	Cross-sectional	Level 4	20
(Duran-Galdo & Mamani-Urrutia, 2021)	Cross-sectional	Level 4	21
(Deisz et al., 2024)	Cross-sectional	Level 4	18
(Foroughi et al., 2022)	Cross-sectional	Level 4	19
(Gallasch et al., 2022)	Cohort	Level 2	20
(Islam et al., 2022)	Cross-sectional	Level 4	20
(Jarso et al., 2023)	Cross-sectional	Level 4	20
(Jamil et al., 2022)	Cross-sectional	Level 4	20
(Kayani et al., 2018)	Cross-sectional	Level 4	22
(Kayani et al., 2020)	Cross-sectional	Level 4	20
(Kayani et al., 2021)	Cross-sectional	Level 4	20
(Kharroubi et al., 2024)	Cross-sectional	Level 4	20
(Lobach et al., 2024)	Quasi-exp.	Level 4	19
(Lun et al., 2018)	Cross-sectional	Level 4	20
(Mahfouz et al., 2024)	Cross-sectional	Level 4	21
(Monserrat-Hernandez et al., 2023)	Cross-sectional	Level 4	20
(Piscoya-Tenorio et al., 2023)	Cross-sectional	Level 4	21
(Pérez et al., 2024)	Cross-sectional	Level 4	21
(Tran et al., 2017)	Cross-sectional	Level 4	20
(Q. Wang et al., 2024)	Cross-sectional	Level 4	20
(Yusuf & Borjac, 2023)	Cross-sectional	Level 4	17
(Zamri & Raman, 2020)	Cross-sectional	Level 4	18
(S. Zhang, 2022)	RCT	Level 1	3/10

Notes: CEBM: Oxford Centre for Evidence-Based Medicine Levels of Evidence. STROBE: Strengthening the Reporting of Observational Studies in Epidemiology (22 items). PEDro: Physiotherapy Evidence Database Scale (10 items).

**Table 3:** Summary of studies on exercise, sport, and physical activity, anxiety, and academic performance.

Author and Year	Subjects	Interv	Anx. Inst.	Anx. Results	Acad. Perf. Inst.	Acad. Perf. Results
(Abbasi et al., 2021)	250	PA	-	not studied	GPA	acad. ↑
(Alaaddin et al., 2021)	400	PA	DASS-21	no effect	GPA	not studied
(Alnofaiey et al., 2023)	2819	PA	HADS	anx. ↓	GPA	acad. ↑
(Brambila-Tapia et al., 2022)	124	PA	GAD-7	no effect	GPA	no effect
(Chacon-Cuberos et al., 2019)	225	PA	MLSQ-SF	anx. ↓	GPA	no effect
(Deisz et al., 2024)	393	Exe.	Ques.	anx. ↓	Ques.	acad. ↑
(Duran-Galdo & Mamani-Urrutia, 2021)	180	PA	SISCO	anx. ↓	-	not studied
(Foroughi et al., 2022)	364	PA	SADS	anx. ↓	GPA	acad. ↑
(Gallasch et al., 2022)	109	PA	STAI	anx. ↓	GPA	not studied
(Islam et al., 2022)	482	PA	Goldb. Scale	anx. ↑	-	not studied
(Jamil et al., 2022)	130	PA	GAD-7	no effect	GPA	not studied
(Jarso et al., 2023)	416	PA	WTAI	anx. ↓	GPA	not studied
(Kayani et al., 2018)	358	PA	Univ. Stress	not studied	GPA	acad. ↑
(Kayani et al., 2020)	418	PA	STAI Y-6	anx. ↓	-	not studied
(Kayani et al., 2021)	305	PA	STAI Y-6	anx. ↓	Ed. Level	not studied
(Kharroubi et al., 2024)	261	PA	GAD-7	anx. ↓	GPA	acad. ↑
(Lobach et al., 2024)	76	PA	EVEA	anx. ↓	GPA	not studied
(Lun et al., 2018)	1119	Exe.	GAD-7	anx. ↓	Ques.	acad. ↑
(Mahfouz et al., 2024)	652	PA	-	not studied	GPA	acad. ↑
(Monserrat-Hernandez et al., 2023)	742	PA	SSI	anx. ↓	-	acad. ↑
(Pérez et al., 2024)	150	PA	DASS-21	no effect	GPA	not studied
(Piscoya-Tenorio et al., 2023)	482	PA	Goldb. Scale	anx. ↑	-	not studied
(Tran et al., 2017)	4184	Exe.	DSM-IV	no effect	-	not studied
(Q. Wang et al., 2024)	413	Sport	Ques.	not studied	GPA, PEP	acad. ↑
(Yusuf & Borjac, 2023)	300	PA	Univ. Stress	anx. ↓	Acad. Perf. Scale	acad. ↑
(Zamri & Raman, 2020)	316	PA	DASS-21	anx. ↓	GPA	no effect
(S. Zhang, 2022)	80	Exe.	SAS	anx. ↓	GPA	acad. ↑

Abbreviations: PA = physical activity; Exe. = exercise; Ques. = questionnaire; HADS = Hospital Anxiety and Depression Scale; DASS-21 = Depression Anxiety and Stress Scale 21; GAD-7 = Generalized Anxiety Disorder Scale; STAI = State-Trait Anxiety Inventory; SADS = Social Avoidance and Distress Scale; WTAI = Westside Test Anxiety Inventory; MLSQ-SF = Motivated Strategies for Learning Questionnaire (Short Form); SISCO = SISCO Academic Stress Inventory; EVEA = Escala de Valoración del Estado de Ánimo; SAS = Self-rating Anxiety Scale; DSM-IV = Diagnostic and Statistical Manual of Mental Disorders (4th ed.); PEP = Perceived Educational Progress; GPA = Grade Point Average. Symbols: ↑ = increase; ↓ = decrease; no effect = no significant change; not studied = variable not analyzed.

## Data Availability

All data generated or analyzed during this study are included in the Supplementary Information Files.

## Supplementary Materials

The following supporting information can be downloaded at: https://www.mdpi.com/article/10.3390/ejihpe15110231/s1, Supplementary File S1: Preferred Reporting Items for Systematic reviews and Meta-Analyses extension for Scoping Reviews (PRISMA-ScR) checklist (Tricco et al., 2018).

## Abbreviations

The following abbreviations are used in this manuscript:

PA	Physical Activity
AP	Academic Performance
HADS	Hospital Anxiety and Depression Scale
DASS-21	Depression Anxiety and Stress Scale 21
GAD-7	Generalized Anxiety Disorder Scale
STAI	State-Trait Anxiety Inventory
SADS	Social Avoidance and Distress Scale
WTAI	Westside Test Anxiety Inventory
MLSQ-SF	Motivated Strategies for Learning Questionnaire (Short Form)
SISCO	SISCO Academic Stress Inventory
EVEA	Escala de Valoración del Estado de Ánimo
SAS	Self-rating Anxiety Scale
DSM-IV	Diagnostic and Statistical Manual of Mental Disorders (4th ed.)
PEP	Perceived Educational Progress
IPAQ	International Physical Activity Questionnaire
GPA	Grade Point Average
